# Omental wrap for salvaging bladder repair: A novel approach

**DOI:** 10.14440/bladder.2024.0058

**Published:** 2025-05-06

**Authors:** Tariq F. Al-Shaiji, Said M. Yaiesh

**Affiliations:** Department of Surgery, Jaber Al Ahmad Hospital, Kuwait City 47780, Kuwait

**Keywords:** Omentum, Cystotomy, Fistula

## Abstract

**Background:**

The bladder represents one of the genitourinary organs most vulnerable to iatrogenic injuries. Ideal repair can be challenging and may require augmentation to fully preserve bladder structure and function. The omentum has been employed as a flap or graft to close bladder defects.

**Objective:**

This study presented a novel approach involving a complete omental wrap for repairing a small-capacity, irreparably damaged bladder following cystotomy in a complex transvesical vesicouterine fistula repair.

**Methods:**

Salvage repair was performed on a severely damaged bladder that had resulted from a chronic vesicouterine fistula, using an omental wrap for augmentation. The omental wrap fully enclosed the bladder to enhance its recovery. The patient had an uneventful post-operative recovery, and follow-up cystography showed a small-capacity bladder with no leakage. Extended follow-up demonstrated resolution of the patient’s symptoms and an improvement in bladder capacity.

**Results:**

This was the first documented application of the omentum as a complete wrap to support an irreparable bladder. The omentum’s mechanical and anti-inflammatory properties provided significant support, leading to a rapid and complete healing of a bladder that initially appeared to have an unfavorable outcome due to extensive inflammation.

**Conclusion:**

The use of omental wrap for bladder repair leverages the omentum’s unique properties to support and augment challenging repairs, resulting in outstanding outcomes and expedited restoration in what would be considered a poor outcome procedure.

## 1. Introduction

Bladder repair is a crucial procedure for a number of urological, obstetrical, gynecological, and colorectal surgeries, as well as during transvesical surgical procedures. The bladder is the genitourinary organ that is most prone to iatrogenic injuries.^1^-^3^ The standard technique for bladder repair involves identifying the site of injury and performing a two-layer, water-tight closure.^1^,^3^ However, this approach is often challenging, especially when the repair is complicated by the loss of tissue or the presence of non-compliant, unamenable tissue that cannot be easily approximated or closed, such as severely inflamed and friable urinary bladder walls.^4^ In these cases, the surgeon’s experience, skill, and occasionally creativity are essential to the substitution, reshaping, and augmentation of the remaining bladder tissue to reconstruct a functional, continent urinary reservoir.

The omentum has been investigated for bladder augmentation by several experimental studies. It, as a flap or a graft, satisfies a number of critical criteria necessary for its successful incorporation in the reconstruction of various organs, including the bladder. Omentocystoplasty has been reportedly successful in animal studies and some clinical reports. The omentum promotes epithelial and muscular regeneration and possesses many essential anti-inflammatory, angiogenic, and sealant properties, making it an ideal graft for closing bladder defects.^5^-^12^

We presented a novel technique that used the greater omentum to completely wrap the bladder and support a challenging bladder repair, which would otherwise have been unsuccessful, following a complicated transvesical vesicouterine fistulectomy. The procedural steps are described, and the post-operative recovery of a functional urinary bladder is detailed.

## 2. Case details

We presented a case of a 54-year-old multiparous perimenopausal woman who had been previously healthy and presented to our clinic with total incontinence. She had no positive surgical or gynecological history, and all her previous births were through normal vaginal delivery. The patient was diagnosed with stress urinary incontinence, which led to the insertion of what we assumed was a tension-free vaginal tape (TVT) by another practitioner in a different country 4 years ago. No groin or lower abdominal surgical scars suggestive of any type of anti-incontinence surgery were noted. Post-TVT, the patient’s incontinence worsened from occasional to total, prompting her to seek help from numerous specialists. Unfortunately, she failed all forms of conservative management, both physical and medical, and the tape was eventually removed.

**Figure 1:**
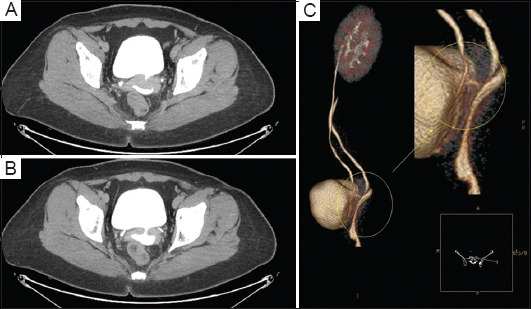
Contrast-enhanced computed tomography images (A and B) in the delayed excretory phase show a full urinary bladder and contrast filling into what was initially believed to be the vaginal vault through a communicating vesicovaginal fistula of around 3 cm in length and of sizable diameter. A digital three-dimensional reconstruction of the excretory phase (C) highlights the fistula.

Initial assessment of the patient revealed leakage upon pelvic examination from the higher vaginal vault. The urodynamic assessment confirmed leakage of a mixed nature. Contrast-enhanced computed tomography (CT), along with three-dimensional reconstruction, confirmed the suspected diagnosis of a vesicovaginal fistula, measuring 3 cm in length, as shown in Figure 1.

### 2.1. Operative steps

**Figure 2:**
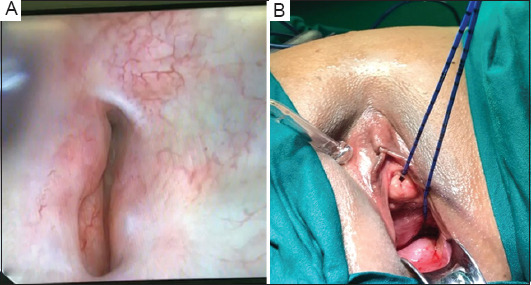
Intraoperative photographs show (A) cystoscopic identification of the vesical end of the fistula at the left posterior urinary bladder wall and (B) retrieval of the ureteric catheter from the uterine cavity after guided cannulation of the fistula, which revealed its other end opening on the lower anterior uterine wall, as confirmed by hysteroscopy.

The patient was scheduled for examination and cystoscopy under general anesthesia, followed by open transvesical vesicovaginal fistula repair. During cystoscopy, we identified a large fistula opening on the left posterior urinary bladder wall near the left ureteric orifice. The fistula exhibited multiple branching daughter tracts that were difficult to cannulate. A speculum examination of the vaginal vault showed no obvious distal fistula opening; however, during bladder filling, fluid gushed from high in the vault, suggesting leakage from the cervix. A hysteroscopy revealed an anterior uterine opening, which was cannulated with a non-traumatic guidewire. The guidewire passed into the bladder, confirming the diagnosis of vesicouterine fistula (VUF). Ureteric catheters were placed bilaterally under fluoroscopic guidance. Figure 2 shows intraoperative photographs of the posterior urinary bladder wall fistula opening and the sequential catheterization after hysteroscopy.

**Figure 3:**
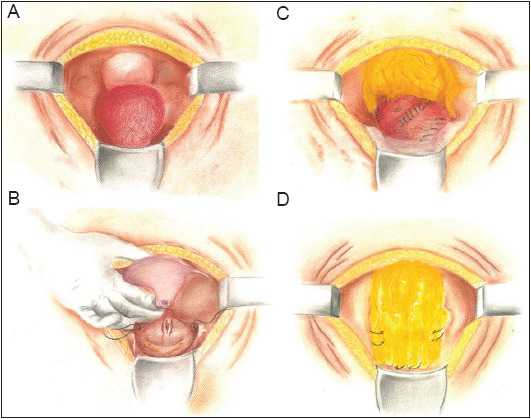
Diagrammatic illustrations of the procedural steps: (A) dissection of layers down to the urinary bladder, (B) fistulectomy and uterine avulsion, (C) bladder repair, leak test, and omental interposition, and (D) final “omental-wrap” of the urinary bladder.

A lower midline incision was made, and a layer-by-layer dissection showed a rather small capacity and chronically inflamed urinary bladder with friable tissue (Figure 3A). Cystotomy was attempted, but due to the friable nature of the bladder tissue, the procedure was halted, and stay sutures were placed for orientation during later repair (Figure 3B). Dissection posterior to the bladder revealed the VUF. While fistulectomy was tried, uterine transection occurred at the level of the uterine neck due to inflammation and adherence of the uterus and anterior vagina to the bladder. This led to a total abdominal hysterectomy without salpingo-oophorectomy under the consultation of a gynecologist who also closed the vaginal vault after the hysterectomy (Figure 3B).

Bladder repair was attempted over a Foley catheter balloon, but difficulties were encountered in approximating the friable bladder walls and maintaining sutures. A leak test performed by instilling saline through an inserted three-way urethral catheter under direct vision yielded a positive result. Further attempts to close the bladder were unsuccessful since sutures failed to hold, and tissue tearing occurred due to suturing (Figure 3C).

### 2.2. Omental wrap technique

The greater omentum was mobilized without the need for gastroepiploic vessel ligation since it was sufficiently long. An interposition flap was initially placed in the posterior plane over the site of the fistula (Figure 3C). Subsequently, by taking advantage of the abundant redundancy of the mobilized greater omentum pedicle, a larger flap was used to cover the superior, lateral, and anterior aspects of the bladder. The flap was secured with single-layer anchoring absorbable sutures at the most inferior aspect of the bladder, fashioned to encapsulate and augment the small bladder and its multiple small defects in a “wrapping” fashion (Figure 3D). A repeat leak test yielded a positive result, though the amount of leakage was significantly reduced compared to the test conducted before the omental wrapping. Postoperatively, the patient was put on minimal continuous bladder irrigation via a three-way urinary catheter. Ureteric catheters were retained and fixed to the urethral catheter to prevent post-operative edema from causing ureteric obstruction, especially given the proximity of the fistula opening to the left ureteric orifice.

### 2.3. Post-operative care and follow-up

Bladder irrigation was discontinued on post-operative day 3, and surgical drains were removed by day 5. The ureteric catheters were also removed. The patient developed no loin pain and maintained normal renal function to her baseline with appropriate urine output.

**Figure 4:**
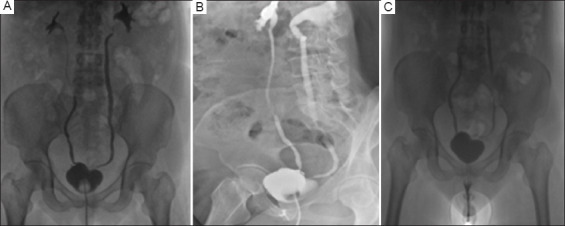
Ascending cystogram shows a small-capacity urinary bladder with Foley’s catheter balloon *in situ* and no leakage on anteroposterior (A) and right lateral oblique images (B); after removal of Foley’s Catheter, micturating cystourethrographic images were obtained (C), and no leak was identified.

An ascending and voiding cystography was planned for post-operative day 21. The study, performed with contrast instillation under gravity control, revealed a small-capacity bladder (the patient tolerated only around 75 mL of contrast, in addition to the catheter balloon volume of 10 cc). No radiological evidence of any leakage was observed, though mild bilateral vesicoureteric reflux was present. The urethral catheter was removed, and the patient voided freely with no post-void residual volume. Figure 4 presents images from the cystography performed, showing no evidence of leakage during bladder filling or after free voiding. The patient was started on low-dose solifenacin to anticipate detrusor overactivity. At follow-up visits every 4 weeks, the dose of solifenacin was increased to address debilitating urinary frequency and urgency, and mirabegron was added 2 weeks later. However, the patient did not experience any episodes of incontinence.

Six weeks after the operation, the patient resumed all her normal daily activities and returned to work. Her obstetrician-gynecologist also examined the vaginal vault closure, confirming complete healing. The patient was given clearance to resume sexual activity with her husband and reported no dyspareunia. She also experienced an improvement in sexual confidence due to the absence of urinary incontinence. Urinary urgency was well controlled, and the frequency of episodes had markedly reduced. The patient reported being able to tolerate larger amounts of fluid intake without the need for frequent voiding, and her voided volume had increased since the removal of the catheter.

A more than 1-year follow-up showed that the patient’s symptoms had significantly improved. Bladder diary assessment and uroflowmetry exhibited an improved functional bladder capacity of 250 mL. The patient reported no episodes of incontinence or leakage. Patient consent and internal department approval have been granted for case discussion and publication (Urology Unit, Amiri Hospital, Kuwait, where the case was assessed and managed).

## 3. Results and discussion

The diagnosis and management of urogenital fistulas pose complex challenges for both urologists and gynecologists. The fistulas are debilitating for patients, can occur at various sites, and result from a spectrum of causes, including iatrogenic injuries. VUFs, in particular, are rare subtypes of urogenital fistulas^1^-^3^,^13^ and account for up to 9% of all urogenital fistulas.^14^

The presentation of VUFs is not always immediately apparent or straightforward, which can complicate diagnosis, even with established classical signs and symptoms.^13^,^15^ Patients typically present with Youssef syndrome, a triad consisting of cyclic hematuria, amenorrhea, and vaginal discharge.^15^ However, other less common presentations, such as urinary incontinence, have also been reported.^13^,^15^-^19^ VUF is practically always of iatrogenic origin, particularly resulting from cesarean deliveries.^18^,^20^ Other reported causes include urinary bladder rupture.^18^

Bladder injuries, when they occur, tend to be initially missed, especially in cases involving foreign body injuries and erosions. Cystoscopy is the definitive diagnostic tool and is virtually always a necessity.^1^,^13^ When bladder injuries are overlooked, they can lead to a multitude of complications, including vesical fistula formation.^21^-^27^ The formation of a fistula allows for the exchange of urine and other substances, depending on the distal component of the fistula. This exchange triggers an inflammatory response in the bladder tissue and may result in urinary tract infections. Prolonged, recurrent insults to the bladder urothelium can ultimately result in tissue friability. Vesical fistulas can also cause urinary incontinence, and, again, depending on their distal component, VUFs may lead to amenorrhea, secondary infertility, abortions, and impaired quality of life, including impacts on sexual function.^1^-^3^,^15^,^28^

In this case report, the patient had no significant obstetric history to suggest a higher insertion of the vesical fistula. The presentation of total incontinence, along with CT findings, led to the diagnosis of vesicovaginal fistula, likely caused by a failed TVT insertion, and intervention was planned accordingly. The intraoperative findings of VUF did not change the surgical approach, which, similar to vesicovaginal fistula repair, involved the excision of the fistula, repair of the urinary bladder and uterus (with or without hysterectomy), and omental interposition.^17^,^28^,^29^ This procedure can be performed transvesically, transperitoneally, transvaginally, or through open, laparoscopic, or robotic-assisted techniques. The need for a hysterectomy depends on the operative findings and specific clinical encounters.^13^,^19^,^28^,^29^ We opted to perform a hysterectomy after the excision of the fistula from the walls of both the severely inflamed and adhered urinary bladder and uterus, which resulted in a complete uterine transection.

After fistulectomy and hysterectomy, attempts to repair the urinary bladder were challenging due to the severe inflammation and friability of the bladder tissue, as well as its small capacity. Traditional methods of urinary bladder repair may not always be feasible in such complicated scenarios, necessitating improvisation and the utilization of alternative modalities for repair in an acute setting.^4^ In our case, after a protracted fistulectomy and additional hysterectomy, bladder closure entailed prompt action. When sutures failed to hold and tore through the bladder tissue, a simple approximation of the remnant bladder wall had to be performed. However, this further decreased the size of the bladder, and despite our efforts, a gap remained, with the bladder tissue remaining weak. An abundant greater omentum, mobilized without ligating the gastroepiploic artery for omental interposition, was considered a suitable adjunct to augment bladder repair. Anchoring sutures were placed around the base of the urinary bladder to create a wrap, effectively encapsulating the bladder in a manner that can be best described as “bagging” or sealing it within the omental covering circumferentially.

The omentum has historically been described and used in various surgical repairs, including urinary bladder repair. It possesses numerous properties that render it an ideal autologous tissue flap or graft. The omentum has a rich vascular and lymphatic supply that promotes the healing of defects. When used in urinary bladder repair, it can augment and enlarge the bladder, facilitate neovascularization and reinnervation, and help maintain bladder tissue integrity and function. Furthermore, it serves as a base template for re-epithelization and muscular regeneration over the covered defect in a relatively short period without the risks of rejection or leakage. The omentum also has anti-inflammatory and sealant properties.^5^-^8^,^12^ Table 1 summarizes the properties of the omentum that make it suitable for bladder augmentation.

**Table 1:** Beneficial properties of the omental graft for bladder repair and rationale for the “omental wrap” approach

Property
Promotes reinnervation
Facilitates neovascularization and angiogenesis
Exhibits anti-inflammatory effects
Resists fibrosis
Is abundant and autologous
Rich vascular and lymphatic supply
Produces VEGF
Transports nutrients and oxygen
Acts as a biological sealant
Supports transitional epithelium and muscle regeneration
Promotes restoration of urinary bladder function
Non-carcinogenic
Hemostatic
Induces minimal or no foreign body response
Structurally strong
Easy to manipulate surgically
Resistant to crystal formation
Tolerant to intraluminal infection

Abbreviation: VEGF: Vascular endothelial growth factor.

Our technique differs from omentocystoplasty, which has been reported in both animal studies and clinical trials. In animal models, omentum has been used to close urinary bladder defects following fistula excision and omental patch incorporation for subtotal cystectomy in rabbits.^5^,^11^ Clinical case reports also described the use of the omentum to close vesicovaginal urinary bladder defects.^5^ In addition, the omentum has been trialed in neurogenic bladder patients with high urinary residual volumes. In these cases, omentovesicopexy successfully reconstructed the urinary bladder through the incorporation of an omental flap into the bladder serosa, resulting in a reduction of urinary residual volume and the restoration of spontaneous urinary continence and control. This success has been attributed to the omentum’s ability to promote the reinnervation of the native bladder tissue.^5^,^8^ Moreover, the omentum has been explored as an option to augment urinary bladders with diminished capacity.^10^ However, it has never been described as a repair template or used in the “wrapped” fashion that we employed to salvage a urinary bladder that was unrepairable by traditional approaches. This method is distinctly different from omentocystoplasty.

The ease of implementing our omental wrap approach and the successful creation of a functional bladder in a case previously deemed intraoperatively irreparable further demonstrate the effectiveness and versatility of the omentum as a material for interposition, augmentation, and substitution. In our case, it functioned as a savior and support for salvage repair. At the inception of the omental wrap technique, its utility was already apparent, as we initially planned to use the omentum for interposition after excising the fistula. However, the technique is theoretically versatile and could potentially be employed as a salvage approach when a bladder is deemed irreparable or when a repair is compromised and likely to fail. This, of course, depends on the availability of the greater omentum, its redundancy to reach deep into the pelvis (with or without ligation of the gastroepiploic vessel), its ability to be used without tension, and the absence of any contraindications, such as prior high abdominal radiation that might impair its properties or reach. The use of the omentum has also been reported to improve and expedite healing and surgical recovery.^5^,^12^ In what was expected to be a prolonged post-operative healing period for the patient, given the leaks evident in the original bladder repair, we were able to remove the surgical drains on post-operative day 5. It is worth noting that the drain collected only a small amount of fluid during its placement, and we never suspected an ongoing leak from the repaired bladder. Thus, the drain could have been removed even earlier, but it was left to stay as a precaution. In addition, the cystography performed before the removal of the urethral catheter showed no evidence of urinary bladder or bladder neck leakage, further supporting our belief that the omental wrap provided timely healing and acted as a sealant to support the repair. The presence of vesicoureteric reflux on the micturating cystogram could not be definitively ascribed to either pre-existing or post-operative sequelae. However, it does not seem to be of a concerning grade or symptomatic, and we plan to follow up with repeat imaging.

The urinary frequency and bladder overactivity that ensued were expected in a small-capacity bladder that had been compromised for an extended period of time due to the long-standing VUF. However, the resolution of these symptoms with the use of anticholinergics and mirabegron, without any leakage, further testified that the repair was successful. The patient has since returned to normal daily activities and expressed satisfaction with both the urinary outcomes and improvement in sexual function – the quality of both being often impaired in patients undergoing VUF repair.^30^

## 4. Conclusion

This case represents the first reported instance of complete bladder repair utilizing the greater omentum as a wrap to support and restore an irreparable bladder. It highlights a novel approach that combines principles of bladder repair and augmentation with the surgical principles of incorporating omental grafts and flaps. A complicated VUF diagnosis and repair should not compromise the surgical outcomes of the procedure. The incorporation of innovative surgical principles of repair from other experiences can significantly improve the results of traditional procedures. Notably, the omentum proves to be especially beneficial in bladder augmentation and repair, as we demonstrated in our case, due to its abundance and several properties that support the healing of bladder defects and maintain bladder functions.

## Data Availability

The data supporting the findings of this study are available from the corresponding author upon reasonable request.
